# Gingival Pigmentation Removal With a High-Power Diode Laser in Non-contact Mode: A Proposed Technique With Two Years of Follow-Up

**DOI:** 10.7759/cureus.31903

**Published:** 2022-11-26

**Authors:** Luciane Hiramatsu Azevedo, Ana Maria de Souza, Patrícia Moreira de Freitas, Ronaldo Tuma, Pedro Cardoso Soares

**Affiliations:** 1 Dentistry, Faculdade de Odontologia, Universidade de São Paulo, São Paulo, BRA; 2 Operative Dentistry, Faculdade de Odontologia, Universidade de São Paulo, São Paulo, BRA

**Keywords:** high-power laser, soft-tissue aesthetics, periodontal management, diode laser, gingival depigmentation

## Abstract

This case report describes the efficacy and advantages of using a high-power diode laser in non-contact mode to remove racial melanin pigmentation from the oral mucosa in an aesthetically sensible area to reduce thermal damage and promote better postoperative results. In the presented case, the lesion was removed under local anesthesia in a single session using high-power diode equipment with circular movements over the lesion (10 seconds maximum for each irradiation cycle, with 30 seconds for thermal relaxation, for a total of two cycles), maintaining an average distance of 2-3 mm from the target tissue without fiber activation (continuous wave, 2 W, delivered by quartz fiber with 300 µ). The patient did not report immediate postoperative pain; however, analgesic use was recorded during the first day after the surgical procedure. The biological response and cosmetic results were satisfactory, with proper lesion removal and adequate cicatrization observed during a two-year follow-up period, without complications or compromise of periodontal tissue esthetics.

## Introduction

Gingival pigmentation is a benign condition that can affect the oral mucosa and can be expressed in different areas of the oral cavity. This condition has different etiologies, and various clinical approaches to treatment are based on the diagnosis. Clinically visualized as the accumulation of dark or brown pigments in the oral mucosa, located in free or attached mucosa, these melanotic macules are derived from melanin production by melanocytes in the basal layer of the epithelium, which are then absorbed by surrounding epithelial cells and connective tissue. The role of melanocytes in the oral mucosa is not yet well understood. Melanin production can be considered physiological or related to neoplastic conditions [1].

Lesions can cause inconvenience to patients, especially in patients with esthetic conditions such as high smile lines, leading to self-image discomfort. Different options to remove oral melanotic macules have been described in the literature, including scalpel surgery, abrasion, electrocautery, cryosurgery, radiosurgery, chemical cauterization, and laser [2].

Diode lasers are a safe and well-described alternative to manage melanotic lesions. Diode lasers (808 nm) target melanin and melanosomes, without causing major damage to the epithelium, connective tissue, and teeth in the area surrounding the site of energy delivery [3], although the technique and parameters utilized can be critical to achieving long-term clinical success and tissue stability. Additionally, studies have demonstrated that lesion recurrence is common during the first years after the procedure, and these recurrences can be reduced when using diode lasers for depigmentation [4].

In the present case report, the authors opted to use a high-power diode laser (808 nm, 2 W) in non-contact mode, without fiber activation, due to lesion characteristics and the localization of the lesion. The combination of pigmentation location and characteristics, along with the surgical technique proposed, contributed as an innovative alternative to manage buccal melanotic pigmentation in esthetically sensitive areas in attached gingiva.

## Case presentation

A 27-year-old woman was referred for the evaluation and treatment of melanotic pigmentation. The patient did not report the presence of previous pathologies or continuous medication use. Furthermore, no substance use, diet, smoking, and other conditions that could lead to extrinsic pigmentation were reported by the patient. The patient reported that the lesion began developing two years prior and had been stable in size and color since then.

**Figure 1:**
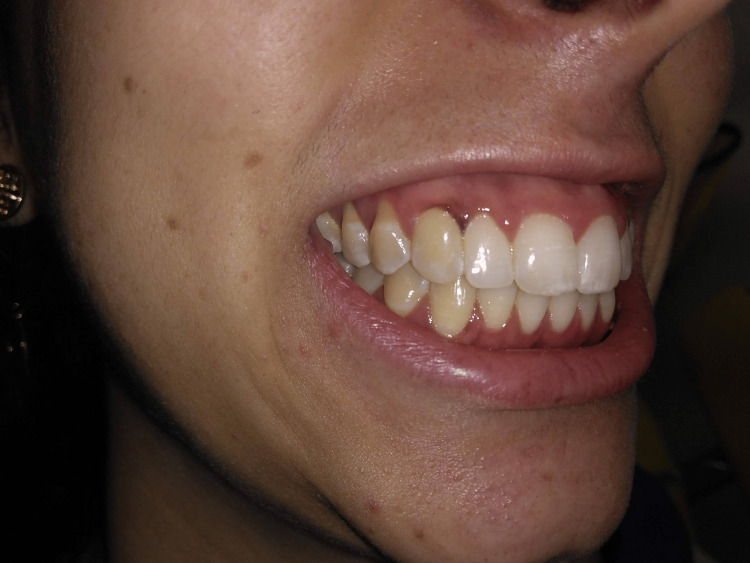
Melanotic pigmentation located in the attached gingiva related to tooth 13, associated with a high smile line, compromising the patient’s esthetic appearance.

The lesion was black/brown in color, located in the attached gingiva, and was limited to the region extending from the anterior to the posterior interdental papilla of tooth 13 (right superior canine). The patient presented a Peeran Pigmentation Index of 1 [5] and class 1 Liebart and Deruelle smile line [6]. The initial aspect of the lesion can be observed in Figure 1.

Informed consent was obtained from the patient before intervention. Surgical removal was indicated due to the lesion compromising the patient’s esthetic appearance.

**Figure 2:**
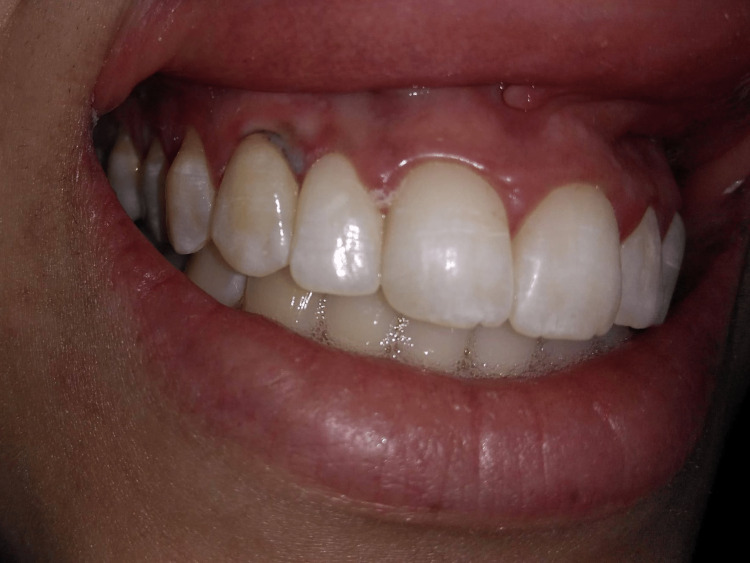
Immediate postoperative aspect after gingival depigmentation with diode laser in non-contact mode (2 W defocused, 2-3 mm).

A high-power diode laser (808 nm, 2 W, Theralase, DMC, São Carlos, SP, Brazil) was used in non-contact mode (defocused), without fiber activation, under local anesthesia. Irradiation was delivered using a 300 µ quartz fiber and kept 2-3 mm away from the lesion surface, in continuous wave mode, and proceeding with quick circular movements in an area for 10 seconds. The endpoint of laser delivery was a sign of blanching on the lesion surface, following which the laser fiber was moved to another area. This procedure allowed gradual irradiation of the entire lesion surface, with two irradiations (10 seconds each for a total of 20 seconds), with an interval of 30 seconds between each irradiation to prevent heat damage. The immediate postoperative aspect can be visualized in Figure 2.

**Figure 3:**
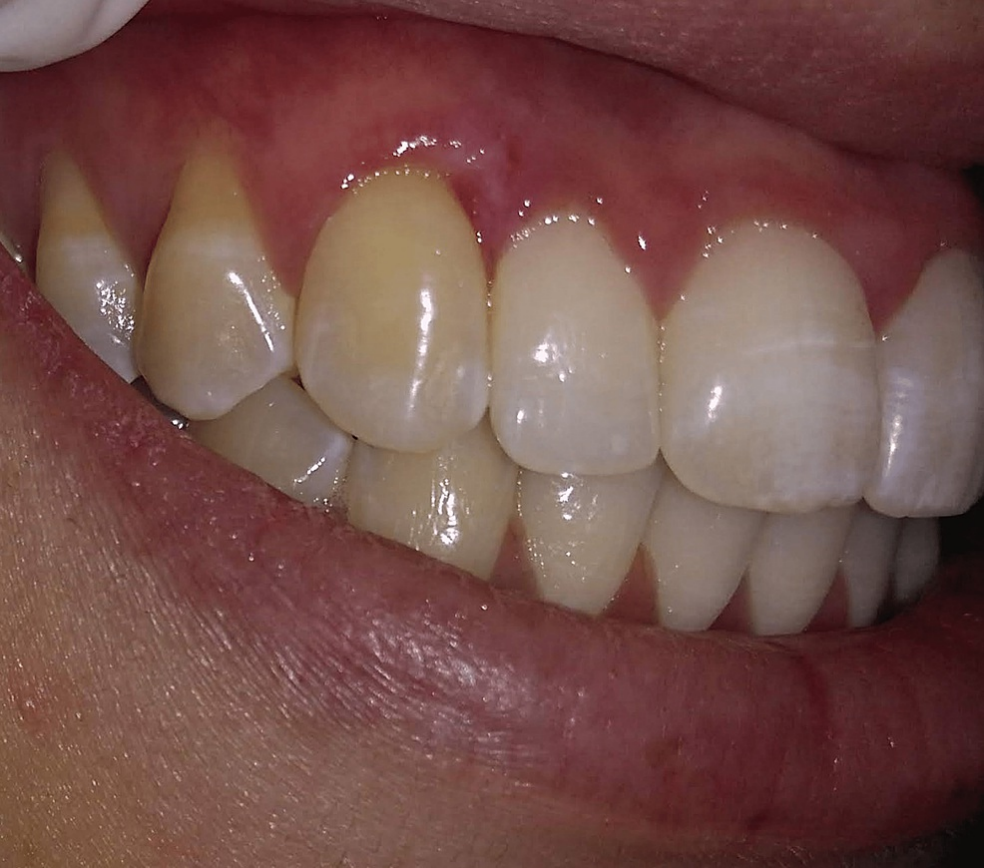
Seven days after attached gingiva depigmentation with diode laser in non-contact mode.

There was no bleeding or any postoperative complications. Immediately after the first laser session, the patient developed slight swelling of the treated area that lasted for three days. No analgesic or anti-inflammatory medication was used. The clinical aspect at seven days postoperatively is shown in Figure 3.

**Figure 4:**
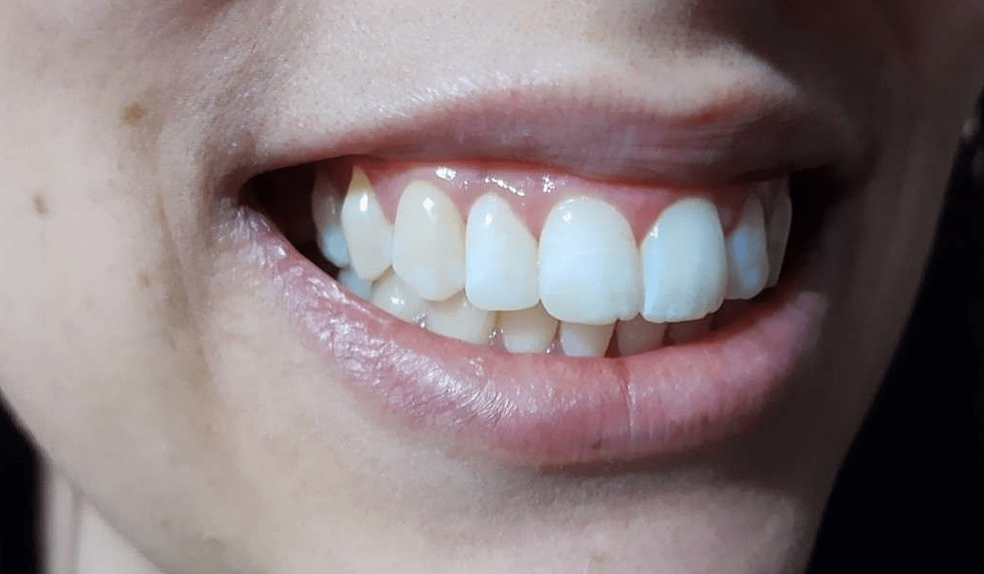
Two-year follow-up image after melanotic pigmentation removal with diode laser in non-contact mode.

The total follow-up period was two years, with no recurrence of lesion or esthetic damage during this period, as observed in Figure 4. The final Peeran Pigmentation Index registered was 0.

## Discussion

Our case report demonstrates the benefits of non-contact, continuous wave, diode laser (808 nm) depigmentation in aesthetically sensible areas in patients with high smile lines. It is also important to observe periodontal stability and overall positive aesthetical outcomes after two years of follow-up.

There are reports of different approaches using different lasers for managing melanic pigmentation, such as CO_2_, erbium, and diode [4]. Diode lasers are highly available when compared to other lasers and have a lower acquiring price. They have a great affinity for pigments, with a greater indication in soft-tissue management. Additionally, they present smaller rates of melanic repigmentation over time compared to other lasers [2,7]. The majority of soft-tissue procedures performed with diode lasers have output power between 2.5 and 1.5 W, continuous wave, with fiber activation, and these parameters create adequate power to cut tissue, with the fiber always placed directly on the tissue surface (contact mode) [8]. On the other hand, the usage of pulsed mode in diode lasers is possible, even though they can lead to gingival fenestrations and bone exposure, caused by the lack of control over temperature rise in gingival tissue [9,10].

Another described option for melanotic pigmentation management is the use of diode lasers in non-contact mode. The “non-contact” technique with diode lasers without fiber activation is not described in the majority of randomized clinical trials and lacks standardization and evidence with prolonged follow-ups [3]. Our study demonstrates that this technique is a great option for melanic pigmentation removal (non-contact, without fiber activation, continuous wave) using parameters available in almost every diode laser equipment, reducing the possibility of undesirable outcomes.

The reduced energy density is the major factor behind adequate depigmentation in the case described, caused by the increased spot size generated by the 2-3 mm distance between the fiber tip and target tissue. The lack of fiber tip activation also plays an important role, allowing direct energy-pigment interaction. This is achievable due to near-infrared wavelength and its affinity for melanin and other pigments, which leads to pigment removal without major damage to surrounding tissues [3,10].

Regarding pain management, diode laser surgery is one of the less invasive and predictable procedures performed on soft tissues in the oral cavity, facilitating visualization during the surgery with minimal bleeding within the intervention site, as well as promoting better immediate esthetic results and patient comfort [11]. This can lead to reduced postoperative pain compared with traditional scalp surgery because sutures are usually not needed. Moreover, lasers can produce photobiomodulation in areas surrounding the surgical site, leading to better pain management [12]. In this case, the Visual Analog Scale (VAS) was used to measure pain during the procedure, immediately after, and one week postoperatively. No pain was reported (VAS = 0) during the procedure, right after, and during the first week. No analgesic use was recorded.

This technique is a safe, quick, previsible, and replicable option to manage racial melanotic pigmentation. More studies evaluating the results obtained with different techniques in aesthetically sensible areas using diode lasers and different populations are needed.

## Conclusions

The use of a high-power diode laser in non-contact mode (defocused) is an effective, bloodless, and safe alternative for removing melanin pigmentation from the oral mucosa. No postoperative complications and pain were reported. This technique generated predictable outcomes when considering periodontal stability and did not cause gingival recession, scars, and recurrence of the lesion during the two-year period of follow-up.
